# Assessment of the Efficacy of Lowering LDL Cholesterol with Rosuvastatin 10 mg in Four Korean Statin Benefit Groups as per ACC/AHA Guidelines (NewStaR4G)

**DOI:** 10.3390/jcm9040916

**Published:** 2020-03-27

**Authors:** Kyung-Jin Kim, Junghan Yoon, Kyung Heon Won, Sang-Wook Lim, In-Ho Chae, Sung Yun Lee, Sang-Wook Kim, Hyo-Soo Kim

**Affiliations:** 1 Division of Cardiology, Department of Internal Medicine, Ewha Womans University Medical Center, Ewha Womans University School of Medicine, Seoul 07985, Korea; 2 Division of Cardiology, Department of Internal Medicine, Yonsei University, Wonju Severance Christian Hospital, Wonju 26426, Korea; 3 Division of Cardiology, Department of Internal Medicine, Seoul Medical Center, Seoul 02053, Korea; 4 Bundang Cha Hospital Cardiovascular Center, Seongnam 13496, Korea; 5 Seoul National University Bundang Hospital, Seoul National University School of Medicine, Seongnam 13620, Korea; 6 Inje University Ilsan Paik Hospital Cardiology, Goyang 10380, Korea; 7 Heart Research Institute Chung-ang University Hospital, Seoul 06973, Korea; 8 Division of Cardiology, Department of Internal Medicine, Seoul National University Hospital, Seoul National University College of Medicine, Seoul 03080, Korea

**Keywords:** Rosuvastatin, cholesterol, LDL, Asian

## Abstract

The American College of Cardiology and American Heart Association (ACC/AHA) guidelines identified four statin benefit groups on the basis of atherosclerotic cardiovascular disease risk reduction and proposed statin therapy by evidence-based intensity. Although these guidelines used randomized controlled trials with hard outcomes as exclusive evidence for its recommendations, a limited number of studies conducted in Asian countries makes its application of treatment strategy, intensity, and statin doses uncertain in these population. This prospective, multicenter study aimed to evaluate the efficacy of rosuvastatin 10 mg in the four statin benefit groups requiring high- or moderate-intensity statin therapy according to the ACC/AHA guidelines in the Korean population. The primary endpoint was percentage reduction in low-density lipoprotein (LDL) cholesterol. Secondary endpoints were percentage reduction in other lipids and achievement of ≥50% reduction in LDL cholesterol. Rosuvastatin 10 mg lowered LDL cholesterol by 61.4 mg/dL, a 44.9% decrease from baseline after eight weeks. Reduction of LDL cholesterol ≥50% was achieved in 46.3% of patients. Rosuvastatin 10 mg was generally well tolerated. In the Korean population, rosuvastatin 10 mg was favorable and tolerant in lowering LDL cholesterol in the four statin benefit groups requiring high- or moderate-intensity statin therapy according to the ACC/AHA guidelines.

## 1. Introduction

The reduction of low-density lipoprotein (LDL) cholesterol levels is one of the cornerstones in the prevention of cardiovascular events [1,2]. Statins, 3-hydroxy-3-methylglutaryl coenzyme reductase inhibitors, are the most effective class of drugs for lowering serum LDL cholesterol levels, and they are the first-line medication to reduce atherosclerotic cardiovascular disease (ASCVD) risk [3,4]. Among the various statins, rosuvastatin, a high-potency third-generation statin [5], has been known to be more effective in lowering LDL cholesterol than simvastatin, atorvastatin, or pravastatin [6,7,8,9,10], and it also induces regression of coronary atherosclerosis [11,12].

The American College of Cardiology and American Heart Association (ACC/AHA) guidelines [3] led to a paradigm shift in cholesterol management by identifying four statin benefit groups on the basis of ASCVD risk reduction and proposing statin therapy using evidence-based intensity without targets [3,13]. These guidelines are also different from previous guidelines in that it used only randomized controlled trials with hard outcomes as exclusive evidence for its recommendations. However, applying these guidelines to an Asian population requires prudence because of the limited number of randomized controlled trials conducted in Asian countries, which results in limited applicability of treatment strategy, intensity, and statin doses [13]. Therefore, further studies using diverse doses of statins in Asian countries are essential.

This study aimed to evaluate the potency of rosuvastatin 10 mg in subjects categorized into the four statin benefit groups requiring high- or moderate-intensity statin therapy according to the ACC/AHA guidelines in South Korea.

## 2. Methods

### 2.1. Study Design

This prospective, multicenter, 8-week study was conducted in 7 centers in South Korea (clinical trials.gov identifier: NCT03903029). All study participants provided written informed consent and the study protocol was approved by institutional review boards at each participating center. We screened subjects who were older than 21 years and who met the criteria of at least one of the four statin benefit groups requiring high- or moderate-intensity statin therapy according to the ACC/AHA guidelines [3]. The four statin benefit groups were as follows: 1) subjects with prior history of clinical ASCVD; 2) subjects with primary elevation of LDL cholesterol ≥190 mg/dL; 3) subjects with diabetes, age 40 to 75 years, and LDL cholesterol 70 to 189 mg/dL; 4) subjects without diabetes, age 40 to 75 years, LDL cholesterol 70 to 189 mg/dL, and with estimated 10-year ASCVD risk ≥7.5%. The main exclusion criteria were: 1) history of significant statin-induced rhabdomyolysis or myopathy; 2) history of a significant hypersensitivity reaction to rosuvastatin; 3) uncontrolled diabetes mellitus (HbA1c >9%); 4) uncontrolled hypertension (systolic blood pressure >190 mmHg or diastolic blood pressure >100 mmHg); 5) current active liver disease (alanine aminotransferase and/or aspartate aminotransferase >2 times the upper limit of normal); 6) chronic kidney disease (serum creatinine clearance <30 mL/min); 7) creatine kinase levels >3 times the upper limit of normal; 8) use of prohibited concomitant therapies; 9) history of malignancy within the last 5 years; 10) women who were pregnant, breast-feeding or of childbearing potential without contraception; and 11) subjects who would take any medication for purposes other than this trial within 30 days after taking this study’s medication. Participating subjects were given rosuvastatin 10 mg daily for 8 weeks after a 4-week wash out period.

### 2.2. Efficacy, Safety, and Stability Assessments

The primary efficacy outcome was the percent reduction in LDL cholesterol from baseline to 8 weeks. Secondary efficacy outcomes were the percent changes from baseline to 8 weeks in total cholesterol, high-density lipoprotein (HDL) cholesterol, triglyceride (TG), non-HDL cholesterol, apolipoprotein B, apolipoprotein A1, and achievement of ≥50% reduction in LDL cholesterol with use of rosuvastatin 10 mg. The percent changes in LDL cholesterol and other lipids from baseline to 8 weeks in the pre-specified four statin benefit groups and in subgroups with diabetes mellitus (DM) and metabolic syndrome (MetS) were also analyzed. The definition of DM was HbA1c ≥6.5%, or a fasting serum glucose level ≥126 mg/dL [14], or self-reported use of an antihyperglycemic drug with a history of DM. The definition of MetS was at least 3 of the following 5 factors: 1) elevated waist circumference (waist circumference ≥90 cm in men, ≥80 cm in women); 2) elevated TG (≥150 mg/dL) or drug treatment for elevated TG; 3) reduced HDL cholesterol (<40 mg/dL in men, <50 mg/dL in women) or drug treatment for reduced HDL cholesterol; 4) elevated fasting glucose ( ≥100 mg/dL) or drug treatment for elevated glucose; 5) elevated blood pressure (systolic blood pressure ≥130 mmHg or diastolic blood pressure ≥85 mmHg) or antihypertensive drug treatment with a history of hypertension [15]. Comparison between patients achieved LDL cholesterol reduction ≥50% or not and was additionally performed after excluding patients with low compliance (<75%).

Safety was evaluated by various laboratory tests, patient-reported adverse symptoms/signs, and the investigators’ observations. In the individual centers, the investigators rated the adverse event (AE) in terms of their intensity (mild, moderate, or severe), seriousness (death or life-threatening events, prolonged hospitalization, and/or disability/incapacitation), and relationship (definitely, probably, possibly, and probably not related) to study medication.

### 2.3. Statistical Analysis

The differences in the baseline characteristics were assessed using Student’s t-test between two groups and one-way analysis of variance (ANOVA) between the four statin benefit groups for continuous variables, and the chi-square test and Fisher’s exact test for categorical variables. The differences in changes in lipid profile were compared using Student’s t-test between two groups and using ANOVA between the four statin benefit groups. For all analyses, a *p* value <0.05 was considered statistically significant. All statistical analyses were conducted using SPSS 22.0 (IBM Co., Armonk, NY, USA). Graphs were drawn with GraphPad Prism 7.0 (GraphPad Software Inc., San Diego, CA, USA).

## 3. Results

### 3.1. Baseline Characteristics

Of the 315 screened subjects, after excluding 15 subjects who withdrew consent (n = 5) or violated the inclusion and exclusion criteria (*n* = 10), 300 subjects were initially assigned the study medication. After an additional exclusion of 58 subjects who withdrew consent (*n* = 18), violated the inclusion or exclusion criteria (*n* = 5), loss of follow up (*n* = 34), and loss of treatment (*n* = 1), 242 subjects were finally analyzed.

The mean age was 66.3 ± 8.2 years and 58.3% of subjects were men. A total of 158 (65.3%) patients had hypertension, 94 (38.8%) patients had DM, 79 (32.6%) patients had MetS, and 95 (39.3%) patients had a history of coronary artery disease (Table 1). Most patients (*n* = 212, 87.6%) were included by clinical ASCVD history and although subjects with clinical ASCVD history had higher prevalence of diabetes mellitus and past history of coronary artery disease than other groups, baseline lipid profiles were not different (Table 1, Table S1).

### 3.2. Efficacy

**Figure 1:**
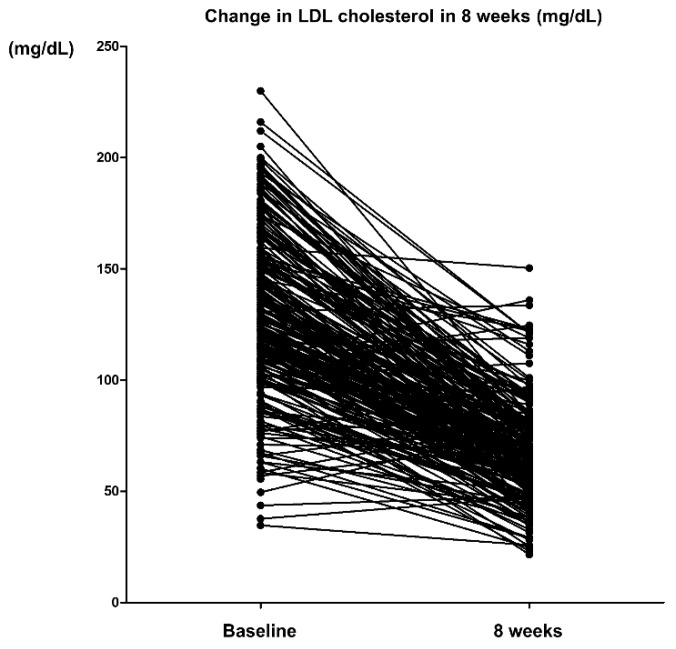
Change in LDL cholesterol in 8 weeks. LDL: low-density lipoprotein.

**Figure 2:**
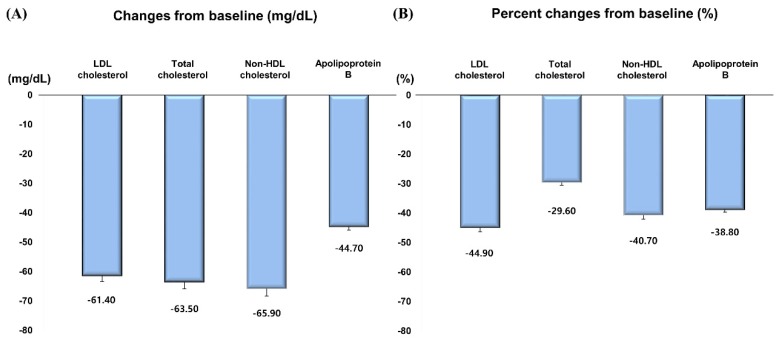
Changes in LDL cholesterol, total cholesterol, non-HDL cholesterol and apolipoprotein B in 8 weeks. (**A**) Changes from baseline. (**B**) Percent changes from baseline. HDL: high-density lipoprotein.

**Figure 3:**
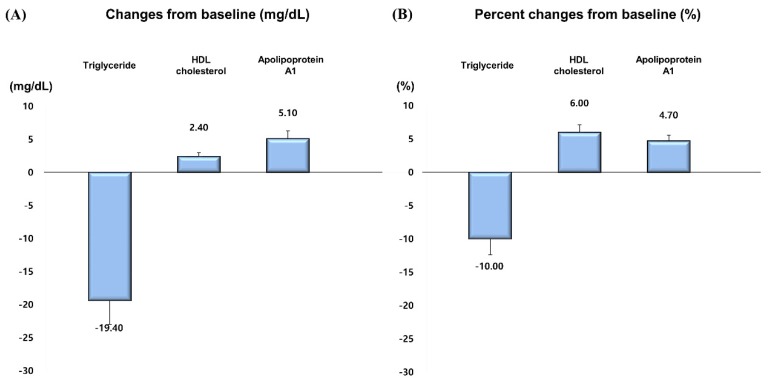
Changes in triglyceride, HDL cholesterol and apolipoprotein A1 in 8 weeks. (**A**) Changes from baseline. (**B**) Percent changes from baseline.

**Figure 4:**
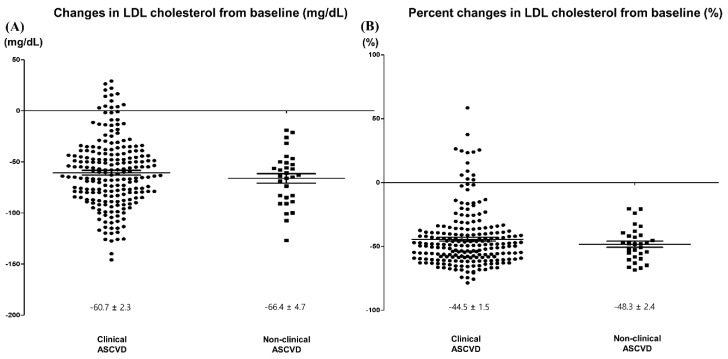
Changes in LDL cholesterol as per the ACC/AHA four statin benefit groups by clinical ASCVD. (**A**) Changes from baseline. (**B**) Percent changes from baseline. ASCVD: clinical atherosclerotic cardiovascular disease.

Rosuvastatin 10 mg reduced LDL cholesterol by 61.4 mg/dL, a 44.9% decrease from baseline at week 8 (Table 2, Table S2, Figure 1 and Figure 2). In terms of the other lipids, rosuvastatin 10 mg reduced total cholesterol by 63.5 mg/dL (29.6%), triglyceride by 19.4 mg/dL (10.0%), non-HDL cholesterol by 65.9 mg/dL (40.7%), apolipoprotein B by 44.7 mg/dL (38.8%), and increased HDL cholesterol by 2.4 mg/dL (6.0%) and apolipoprotein A1 by 5.1 mg/dL (4.7%) (Table 2, Table S2, Figure 2 and Figure 3). Reduction rate was not significantly different by the pre-specified four statin benefit groups (Table 2, Table S2, Figure 4, Figure S1).

Achievement of ≥50% reduction in LDL cholesterol by rosuvastatin 10 mg was 46.3% (112/242) in all patients. A comparison of subjects who reached 50% LDL cholesterol reduction versus others was performed (Table 3) in patients whose compliance was greater than 75%. The prevalence of comorbidities and rates of inclusion criteria were not significantly different between groups, except history of dyslipidemia. The subjects who achieved LDL reduction ≥50% (109/237, 46.0%) had higher baseline lipid values, except triglyceride (Table 3), and showed higher reduction in total cholesterol, triglyceride, non-HDL cholesterol and apolipoprotein B (Table 4). Meanwhile, the subjects who achieved LDL reduction ≥50% showed a lower increase in apolipoprotein A1 and lower but not significant increase in HDL cholesterol (Table 4).

Regarding underlying DM and MetS, the efficacy of rosuvastatin 10 mg was not significantly different between subjects with or without DM (Table 5). However, in MetS, an increase in HDL cholesterol was higher in subjects with MetS (9.5% versus 4.3%, *p* = 0.030) than without (Table 6).

### 3.3. Safety

The adherence of all patients at 8 weeks was 94.7% (95% confidence interval (CI) of 92.6 to 96.7). No event of consecutive elevation ≥3 times the upper normal limits in alanine aminotransferase or aspartate aminotransferase occurred (Table 7). Two patients (0.7%) were reported to have elevations ≥5 times the upper limit of normal of creatine kinase. Both patients reported performing heavy muscular activities the day before the visit and normalized without treatment. Except the increased creatine kinase level, the reported drug-related AEs were limited to generalized itching, urticaria, and dry mouth and the symptoms improved without treatment. Other serious AEs were cerebral infarction in two patients, although these were not considered drug-related AEs.

## 4. Discussion

This study sought to evaluate the efficacy and safety of rosuvastatin 10 mg in the treatment of the four statin benefit groups requiring high- or moderate-intensity statin therapy according to the ACC/AHA guidelines in the Korean population.

In this study, we confirmed rosuvastatin 10 mg was favorable and tolerant in the four statin benefit groups for managing LDL cholesterol and other lipids including total cholesterol, triglyceride, HDL cholesterol, non-HDL cholesterol, apolipoprotein B, and apolipoprotein A1 in the Korean population. Moreover, rosuvastatin 10 mg was acceptable in terms of achieving LDL cholesterol reduction ≥50%.

Since the first guideline recommending treatments for lowering cholesterol was released by the National Cholesterol Education Program (NCEP) in 1998 [16], the idea that lowering LDL cholesterol levels is one of the cornerstones in the prevention of cardiovascular events has been accepted in all future recommendations. This first guideline, the Adult Treatment Panel (ATP) I, defined target values for LCL cholesterol less than 130 mg/dL to 160mg/dL according to estimated coronary risk [16] and following this, ATP II [17] and ATP III [2] set a new goal of <100mg/dL for the highest risk groups.

In 2013, there was a paradigm shift by new guidelines on the management of blood cholesterol released by ACC/AHA [3]. These guidelines were differentiated from previous guidelines by using only randomized controlled trials with hard outcomes as evidence, proposing statin therapy using evidence-based intensity without targets, and identifying four statin benefit groups on the basis of ASCVD risk reduction. Even though these guidelines were updated in 2018 [18], the mainstream ideas that emphasizing attention on the reduction of ASCVD risk, focusing on the intensity of the drug rather than the target value, and identifying patient groups in whom statin therapy is recommended based on the four statin benefit groups, was maintained. Therefore, consideration of this new perspective in clinical fields is worth applying.

Although there has been previous studies which evaluated the efficacy and safety of statins in the Asian population, due to the limited number of randomized controlled trials with hard outcomes conducted in Asian countries which used exclusive evidence for these guidelines, and moreover, limited studies conducted within patients categorized into the four statin benefit groups, the suitability of applying this guideline to Asian populations was uncertain.

In this study, the percent reduction rate of LDL cholesterol was 44.9% and the target LDL achievement rate (≥50% reduction in LDL cholesterol) by rosuvastatin 10 mg was 46.3%. In previous reports, LDL cholesterol reduction rate by rosuvastatin 10 mg was 44.6% to 47.5% [10,19,20]. However, the study populations of these studies were subjects with primary hypercholesterolemia or those who qualified for lipid-lowering therapy, not the four statin benefit groups identified by the ACC/AHA guidelines, which limits the direct comparison. One previous study reported a ≥50% rate of reduction in LDL cholesterol in the ASCVD group as defined by the ACC/AHA guidelines as 57% and 71% with rosuvastatin 20 mg and 40 mg, respectively [21]. The 46.3% achievement rate of our study was lower than this report. However, considering the lower dose of our study protocol, achieving ≥50% reduction in LDL cholesterol in almost half of the study subjects might be considered acceptable. Moreover, it should be considered that increasing the dose of the statin contributes a limited additional reduction rate and may result in a higher rate of side effects [22,23,24].

Meanwhile, these previous studies were mostly conducted in Western countries, and few clinical trials carried out in Asia demonstrated the efficacy and safety of statins [25]. A few clinical studies were conducted in Japan, and the reduction rates of LDL cholesterol with rosuvastatin 10 mg were 49.2% [26] and 49.7% [27], which were similar to our study. In Korea, our group recently reported the LDL reduction rate with rosuvastatin 10 mg of 52% [24]. However, the study population of these studies were subjects with familial hypercholesterolemia [26] or hyper LDL cholesterolemia [27], not subjects within the four statin benefit groups as defined by the ACC/AHA guideline. Besides these studies, there were several reports that the plasma concentration of rosuvastatin and metabolites were higher in Asian subjects than Caucasian subjects, and body weight differences contributed to the pharmacokinetic differences (less than 10%) [25,28], but data from Asian populations are still limited.

We expect that our study may give additional evidence of the efficacy of rosuvastatin 10 mg in the four statin benefit groups defined by the ACC/AHA guidelines, especially in Asian populations.

## 5. Conclusions

In conclusion, this study supports the efficacy and safety of rosuvastatin 10 mg in the four statin benefit groups focused on ASCVD risk reduction as identified by the ACC/AHA guidelines in the Korean population. Moreover, in the Korean population, rosuvastatin 10 mg is acceptable to achieve a 50% reduction in LDL cholesterol.

## Figures and Tables

**Table 1:** Baseline characteristics as per The American College of Cardiology and American Heart Association (ACC/AHA) guidelines of the four statin benefit groups, by clinical atherosclerotic cardiovascular disease (ASCVD).

	Total (*n* = 242)	Clinical ASCVD (*n* = 212)	Composite of Groups 2, 3 and 4 of the Four Statin Benefit Groups (*n* = 30)	*p* Value
**Demographic**				
Age, years	66.3 ± 8.2	66.3 ± 8.3	66.0 ± 7.5	0.848
Male, *n* (%)	141 (58.3)	128 (60.4)	13 (43.3)	0.076
BMI, kg/m^2^	25.4 ± 4.2	25.2 ± 4.1	26.5 ± 4.7	0.129
Current smoker, *n* (%)	33 (13.6)	29 (13.7)	4 (13.3)	>0.999
Hypertension, *n* (%)	158 (65.3)	136 (64.2)	22 (73.3)	0.323
Diabetes mellitus, *n* (%)	94 (38.8)	72 (82.3)	22 (73.3)	<0.001
Dyslipidemia, *n* (%)	148 (61.2)	127 (59.9)	21 (70.0)	0.288
Metabolic syndrome, *n* (%)	79 (32.6)	71 (69.2)	8 (26.7)	0.456
Past history of CAD, *n* (%)	95 (39.3)	94 (44.3)	1 (3.3)	<0.001
Congestive heart failure	4 (1.7)	4 (1.9)	0	>0.999
Stroke	8 (3.3)	8 (3.8)	0	0.601
Peripheral artery occlusive disease	9 (3.7)	9 (4.2)	0	0.606
**Washout information, *n* (%)**				
Atorvastatin	9 (3.7)	7 (3.3)	2 (6.7)	0.309
Pitavastatin	1 (0.4)	1 (0.5)	0	>0.999
Simvastatin	0 (0)	0	0	
Pravastatin	2 (0.8)	2 (0.9)	0	>0.999
Rosuvastatin	6 (2.5)	6 (2.8)	0	>0.999
**Baseline lipid profile**				
LDL cholesterol, mg/dL	128.9 ± 26.5	128.0 ± 37.1	135.3 ± 32.1	0.304
Total cholesterol mg/dL	203.0 ± 41.7	202.1 ± 42.1	209.0 ± 38.1	0.397
Triglycerides, mg/dL	130.9 ± 54.9	129.9 ± 53.8	137.8 ± 62.4	0.460
HDL cholesterol, mg/dL	50.1 ± 12.7	50.0 ± 13.0	51.3 ± 10.4	0.602
Non-HDL cholesterol, mg/dL	152.8 ± 38.7	152.1 ± 39.1	157.8 ± 36.2	0.459
Apolipoprotein B, mg/dL	113.0 ± 22.3	113.2 ± 22.5	111.9 ± 21.4	0.773
Apolipoprotein A1, mg/dL	132.4 ± 22.1	131.8 ± 22.4	136.0 ± 20.1	0.331

Variables are presented as mean (SD) or *n* (%). SD, standard deviation; BMI, body mass index; LDL, low-density lipoprotein; CAD, coronary artery disease; HDL, high-density lipoprotein.

**Table 2:** Changes in lipid profiles as per ACC/AHA guidelines for the four statin benefit groups, by clinical atherosclerotic cardiovascular disease (ASCVD).

	Total (*n* = 242)	Clinical ASCVD (*n* = 212)	Composite of Groups 2, 3 and 4 of the Four Statin Benefit Groups (*n* = 30)	*p* Value
**Absolute change**				
LDL cholesterol, mg/dL	−61.4 ± 2.1	−60.7 ± 2.3	−66.4 ± 4.7	0.382
Total cholesterol, mg/dL	−63.5 ± 2.4	−63.0 ± 2.6	−67.3 ± 5.8	0.550
Triglycerides, mg/dL	−19.4 ± 3.6	−17.7 ± 3.9	−31.3 ± 9.3	0.219
HDL cholesterol, mg/dL	2.4 ± 0.6	2.1 ± 0.6	4.2 ± 1.8	0.237
Non−HDL cholesterol, mg/dL	−65.9 ± 2.4	−65.1 ± 2.6	−71.4 ± 5.2	0.374
Apolipoprotein B, mg/dL	−44.7 ± 1.3	−44.8 ± 1.4	−44.0 ± 3.5	0.822
Apolipoprotein A1, mg/dL	5.1 ± 1.2	5.3 ± 1.3	3.5 ± 3.6	0.632
**Percent change**				
LDL cholesterol, %	−44.9 ± 1.4	−44.5 ± 1.5	−48.3 ± 2.4	0.357
Total cholesterol, %	−29.6 ± 1.0	−29.3 ± 1.1	−31.2 ± 2.2	0.541
Triglycerides, %	−10.0 ± 2.4	−9.1 ± 2.6	−16.3 ± 5.5	0.318
HDL cholesterol, %	6.0 ± 1.1	5.5 ± 1.2	9.6 ± 3.5	0.234
Non-HDL cholesterol, %	−40.7 ± 1.3	−40.2 ± 1.5	−44.3 ± 2.4	0.299
Apolipoprotein B, %	−38.8 ± 0.9	−38.8 ± 1.0	−38.5 ± 2.5	0.921
Apolipoprotein A1, %	4.7 ± 0.9	4.9 ± 1.0	3.8 ± 2.6	0.698

Variables are presented as the means ± SE. SE, standard error.

**Table 3:** Comparison of subjects who reached 50% LDL cholesterol reduction versus others.

	Total (*n* = 237)	LDL-C Reduction <50% (*n* = 128)	LDL-C Reduction ≥50% (*n* = 109)	*p* Value
**Demographic**				
Age, years	66.2 ± 8.2	67.1 ± 7.2	65.1 ± 9.2	0.060
Male, *n* (%)	138 (58.2)	77 (60.2)	61 (56.0)	0.514
BMI, kg/m^2^	25.4 ± 4.2	25.6 ± 4.2	25.2 ± 4.2	0.556
**Inclusion criteria**				0.273
Group 1 (ASCVD)	208 (87.8)	112 (87.5)	96 (88.0)	
Group 2 (LDL ≥190)	3 (1.2)	0	3 (2.8)	
Group 3 (DM)	19 (8.0)	12 (9.4)	7 (6.4)	
Group 4 (10yr risk ≥7.5%)	7 (3.0)	4 (3.1)	3 (2.8)	
Current smoker, *n* (%)	33 (13.9)	18 (14.1)	15 (13.8)	0.947
Hypertension, *n* (%)	156 (65.8)	81 (63.3)	75 (68.8)	0.371
Diabetes mellitus, *n* (%)	93 (39.2)	50 (39.1)	43 (39.4)	0.952
Dyslipidemia, *n* (%)	146 (61.6)	62 (48.4)	84 (77.1)	<0.001
Metabolic syndrome, *n* (%)	79 (33.3)	43 (33.6)	36 (33.0)	0.927
Past history of CAD, *n* (%)	92 (38.8)	43 (33.6)	49 (45.0)	0.074
Congestive heart failure	4 (1.7)	2 (1.6)	2 (1.8)	>0.999
Stroke	7 (3.0)	3 (2.3)	4 (3.7)	0.706
Peripheral artery occlusive disease	9 (3.8)	6 (4.7)	3 (2.8)	0.512
**Washout information, *n* (%)**				
Atorvastatin	9 (3.8)	7 (5.5)	2 (1.8)	0.184
Pitavastatin	1 (0.4)	1 (0.8)	0	>0.999
Simvastatin	0	0	0	
Pravastatin	2 (0.8)	0	2 (1.8)	0.210
Rosuvastatin	6 (2.5)	5 (3.9)	1 (0.9)	0.222
**Baseline lipid profile**				
LDL cholesterol, mg/dL	129.4 ± 36.6	119.0 ± 35.5	141.7 ± 34.1	<0.001
Total cholesterol mg/dL	203.6 ± 41.8	192.0 ± 40.7	217.3 ± 39.0	<0.001
Triglycerides, mg/dL	131.5 ± 55.1	131.1 ± 58.0	132.0 ± 51.8	0.904
HDL cholesterol, mg/dL	50.1 ± 12.8	48.4 ± 12.3	52.1 ± 13.1	0.026
Non-HDL cholesterol, mg/dL	153.5 ± 38.7	143.6 ± 38.0	165.2 ± 36.4	<0.001
Apolipoprotein B, mg/dL	113.3 ± 22.3	109.8 ± 20.0	117.5 ± 24.2	0.008
Apolipoprotein A1, mg/dL	132.0 ± 22.1	129.3 ± 20.7	135.2 ± 23.4	0.043

Variables are presented as mean (SD) or *n* (%).

**Table 4:** Changes in lipid profiles of subjects who reached 50% LDL cholesterol reduction versus others.

	Total (*n* = 237)	LDL-C Reduction <50% (*n* = 128)	LDL-C Reduction ≥50% (*n* = 109)	*p* Value
**Absolute change**				
LDL cholesterol, mg/dL	−61.5 ± 2.2	−41.4 ± 2.4	−85.0 ± 2.2	<0.001
Total cholesterol, mg/dL	−63.5 ± 2.4	−42.1 ± 2.7	−88.7 ± 2.5	<0.001
Triglycerides, mg/dL	−19.1 ± 3.7	−9.3 ± 5.6	−30.7 ± 4.4	0.004
HDL cholesterol, mg/dL	2.4 ± 0.6	3.0 ± 0.7	1.6 ± 0.9	0.218
Non-HDL cholesterol, mg/dL	−65.9 ± 2.4	−45.1 ± 2.8	−90.3 ± 2.5	<0.001
Apolipoprotein B, mg/dL	−44.7 ± 1.3	−35.5 ± 1.7	−55.5 ± 1.6	<0.001
Apolipoprotein A1, mg/dL	5.2 ± 1.3	7.8 ± 1.9	2.2 ± 1.5	0.027
**Percent change**				
LDL cholesterol, %	−44.7 ± 1.4	−31.7 ± 1.9	−59.9 ± 0.6	<0.001
Total cholesterol, %	−29.4 ± 1.0	−20.3 ± 1.3	−40.2 ± 0.7	<0.001
Triglycerides, %	−9.7 ± 2.4	−2.4 ± 3.8	−18.2 ± 2.8	0.001
HDL cholesterol, %	6.0 ± 1.1	7.3 ± 1.5	4.5 ± 1.8	0.233
Non-HDL cholesterol, %	−40.4 ± 1.3	−28.7 ± 1.9	−54.2 ± 0.7	<0.001
Apolipoprotein B, %	−38.6 ± 0.9	−31.6 ± 1.3	−46.8 ± 0.7	<0.001
Apolipoprotein A1, %	4.8 ± 1.0	7.2 ± 1.5	2.1 ± 1.1	0.007

Variables are presented as the means ± SE.

**Table 5:** Percentage changes in lipid profiles by diabetes mellitus.

	Total (*n* = 242)	Non-Diabetic (*n* = 148)	Diabetic (*n* = 94)	*p* Value
**Absolute change**				
LDL cholesterol, mg/dL	−61.4 ± 2.1	−61.3 ± 2.8	−61.6 ± 3.2	0.944
Total cholesterol, mg/dL	−63.5 ± 2.4	−63.9 ± 3.2	−62.8 ± 3.4	0.809
Triglycerides, mg/dL	−19.4 ± 3.6	−15.4 ± 5.2	−25.7 ± 4.4	0.166
HDL cholesterol, mg/dL	2.4 ± 0.6	1.9 ± 0.8	3.1 ± 0.9	0.327
Non-HDL cholesterol, mg/dL	−65.9 ± 2.4	−65.9 ± 3.2	−65.8 ± 3.4	0.996
Apolipoprotein B, mg/dL	−44.7 ± 1.3	−44.5 ± 1.8	−45.2 ± 1.8	0.792
Apolipoprotein A1, mg/dL	5.1 ± 1.2	4.4 ± 1.7	6.1 ± 1.8	0.505
**Percent change**				
LDL cholesterol, %	−44.9 ± 1.4	−44.6 ± 1.8	−45.4 ± 2.1	0.780
Total cholesterol, %	−29.6 ± 1.0	−29.3 ± 1.3	−30.0 ± 1.4	0.712
Triglycerides, %	−10.0 ± 2.4	−7.3 ± 2.1	−14.3 ± 3.7	0.153
HDL cholesterol, %	6.0 ± 1.1	5.0 ± 1.5	7.6 ± 1.7	0.271
Non-HDL cholesterol, %	−40.7 ± 1.3	−40.0 ± 1.8	−41.7 ± 1.8	0.537
Apolipoprotein B, %	−38.8 ± 0.9	−37.9 ± 1.3	−40.1 ± 1.2	0.264
Apolipoprotein A1, %	4.7 ± 0.9	4.1 ± 1.3	5.7 ± 1.4	0.408

Variables are presented as the means ± SE.

**Table 6:** Percentage changes in lipid profiles by metabolic syndrome.

	Total (*n* = 242)	Non-MetS (*n* = 163)	MetS (*n* = 79)	*p* Value
**Absolute change**				
LDL cholesterol, mg/dL	−61.4 ± 2.1	−63.6 ± 2.4	−57.8 ± 4.2	0.270
Total cholesterol, mg/dL	−63.5 ± 2.4	−63.6 ± 2.6	−63.3 ± 4.9	0.950
Triglycerides, mg/dL	−19.4 ± 3.6	−13.2 ± 3.1	−32.2 ± 9.0	0.047
HDL cholesterol, mg/dL	2.4 ± 0.6	1.8 ± 0.8	3.6 ± 0.8	0.099
Non-HDL cholesterol, mg/dL	−65.9 ± 2.4	−65.4 ± 2.6	−66.8 ± 5.0	0.796
Apolipoprotein B, mg/dL	−44.7 ± 1.3	−43.8 ± 1.6	−48.8 ± 2.2	0.028
Apolipoprotein A1, mg/dL	5.1 ± 1.2	3.7 ± 1.7	8.1 ± 1.6	0.094
**Percent change**				
LDL cholesterol, %	−44.9 ± 1.4	−46.5 ± 1.5	−41.7 ± 2.8	0.133
Total cholesterol, %	−29.6 ± 1.0	−29.9 ± 1.0	−28.8 ± 2.1	0.650
Triglycerides, %	−10.0 ± 2.4	−8.3 ± 2.5	−13.6 ± 5.0	0.290
HDL cholesterol, %	6.0 ± 1.1	4.3 ± 1.4	9.5 ± 1.9	0.030
Non-HDL cholesterol, %	−40.7 ± 1.3	−42.0 ± 1.4	−38.0 ± 2.8	0.212
Apolipoprotein B, %	−38.8 ± 0.9	−38.0 ± 1.2	−40.3 ± 1.3	0.181
Apolipoprotein A1, %	4.7 ± 0.9	3.5 ± 1.3	7.2 ± 1.3	0.071

Variables are presented as the means ± SE.

**Table 7:** Safety endpoints: adverse events.

Adverse experience	(n = 300)
All adverse events, n (%)^a^	32 (10.7)
Drug-related, n (%)^a^	6 (2.0)
Definitely, n (%)	0
Probably, n (%)	1 (0.3)
Possibly, n (%)	4 (1.3)
Probably not related, n (%)	4 (1.3)
Serious, n (%)	2 (0.7)
Death or life-threatening, n (%)	0
Prolonged hospitalization, n (%)	0
Disability/incapacitation, n (%)	0
Liver function tests ≥ 3 × ULN	
Alanine aminotransferase, n (%)	0
Aspartate aminotransferase, n (%)	0
Creatine kinase ≥ 5 × ULN, n (%)	2 (0.7)

AE, adverse event; ULN, upper limit of normal, ^a^ Considered by the investigator to be related to the study drug.

## Supplementary Materials

The following are available online at https://www.mdpi.com/2077-0383/9/4/916/s1, Figure S1: Changes in LDL cholesterol per ACC/AHA 4 statin benefit groups, (A) Changes from baseline. (B) Percent changes from baseline. LDL, low density lipoprotein, Table S1: Baseline characteristics per ACC/AHA guidelines 4 statin benefit groups, Table S2: Changes in lipid profiles per ACC/AHA guidelines 4 statin benefit groups.
