# Supplementary material for: Assessment of the Efficacy of Lowering LDL Cholesterol with Rosuvastatin 10 mg in Four Korean Statin Benefit Groups as per ACC/AHA Guidelines (NewStaR4G)
Source: J Clin Med. 2020 Mar 27;9(4):916. doi: 10.3390/jcm9040916 (PMC7230727; doi:10.3390/jcm9040916)
Supplement: Supplementary file 1 [file jcm-09-00916-s001.pdf]

Supplementary Table 1. Baseline characteristics per ACC/AHA guidelines 4 statin benefit groups.

	Total (n = 242)	Clinical ASCVD (n = 212)	Primary LDL-C ≥190 mg/dL (n = 3)	Diabetes 40-75 yr. LDL-C 70-189 mg/dL (n = 20)	No diabetes 40-75 yr. LDL-C 70-189 mg/dL, 10 year risk ≥7.5% (n = 7)	*P* value
Demographic						
Age, years	66.3 ± 8.2	66.3 ± 8.3	68.0 ± 13.2	65.8 ± 6.8	65.7 ± 7.7	0.972
Male, n (%)	141 (58.3)	128 (60.4)	0	10 (50.0)	3 (42.9)	0.121
BMI, kg/m^2	25.4 ± 4.2	25.2 ± 4.1	24.7 ± 4.0	26.2 ± 3.9	28.2 ± 6.8	0.254
Current smoker, n (%)	33 (13.6)	29 (13.7)	0	3 (15.0)	1 (14.3)	0.934
Hypertension, n (%)	158 (65.3)	136 (64.2)	3 (100)	17 (13.1)	2 (4.6)	0.024
Diabetes mellitus, n (%)	94 (38.8)	72 (82.3)	1 (33.3)	20 (100)	1 (14.3)	<0.001
Dyslipidemia, n (%)	148 (61.2)	127 (59.9)	3 (100)	11 (55.0)	7 (4.3)	0.068
Metabolic syndrome, n (%)	79 (32.6)	71 (69.2)	0	4 (20.0)	4 (57.1)	0.210
Past history of CAD, n (%)	95 (39.3)	94 (44.3)	0	1 (5.0)	0	<0.001
Congestive heart failure	4 (1.7)	4 (1.9)	0	0	0	>0.999
Stroke	8 (3.3)	8 (3.8)	0	0	0	>0.999
Peripheral artery occlusive disease	9 (3.7)	9 (4.2)	0	0	0	>0.999
Washout information, n (%)						
Atorvastatin	9 (3.7)	7 (3.3)	0	0	2 (28.6)	0.066
Pitavastatin	1 (0.4)	1 (0.5)	0	0	0	>0.999
Simvastatin	0 (0)	0	0	0	0	
Pravastatin	2 (0.8)	2 (0.9)	0	0	0	>0.999
Rosuvastatin	6 (2.5)	6 (2.8)	0	0	0	>0.999
Baseline lipid profile						
LDL cholesterol, mg/dL	128.9 ± 26.5	128.0 ± 37.1	189.0 ± 8.2	137.0 ± 22.8	107.5 ± 31.0	0.008
Total cholesterol mg/dL	203.0 ± 41.7	202.1 ± 42.1	261.3 ± 35.1	208.3 ± 31.8	188.6 ± 39.1	0.066
Triglycerides, mg/dL	130.9 ± 54.9	129.9 ± 53.8	118.7 ± 29.0	126.3 ± 31.2	179.1 ± 113.2	0.123
HDL cholesterol, mg/dL	50.1 ± 12.7	50.0 ± 13.0	57.0 ± 15.1	52.5 ± 9.5	45.3 ± 10.0	0.457
Non-HDL cholesterol, mg/dL	152.8 ± 38.7	152.1 ± 39.1	204.3 ± 20.2	155.8 ± 29.6	143.3 ± 45.5	0.115
Apolipoprotein B, mg/dL	113.0 ± 22.3	113.2 ± 22.5	137.3 ± 14.6	107.3 ± 16.7	114.0 ± 29.4	0.182
Apolipoprotein A1, mg/dL	132.4 ± 22.1	131.8 ± 22.4	133.7 ± 27.6	135.5 ± 18.4	138.7 ± 24.7	0.780

Variables are presented as mean (SD) or n (%). SD, standard deviation; ASCVD, atherosclerotic cardiovascular disease; BMI, body mass index; LDL-C, low-density lipoprotein cholesterol; CAD, coronary artery disease; HDL, high-density lipoprotein.

Supplementary Table 2. Changes in lipid profiles per ACC/AHA guidelines 4 statin benefit groups.

	**Total (n = 242)**	**Clinical ASCVD (n = 212)**	**Primary LDL-C ≥190 mg/dL (n = 3)**	**Diabetes 40-75 yr. LDL-C 70-189 mg/dL (n = 20)**	**No diabetes 40-75 yr. LDL-C 70-189 mg/dL, 10 year risk ≥7.5% (n = 7)**	***P* value**
Absolute change						
LDL cholesterol, mg/dL	-61.4 ± 2.1	-60.7 ± 2.3	-109.3 ± 8.8	-65.2 ± 4.1	-51.3 ± 11.3	0.062
Total cholesterol, mg/dL	-63.5 ± 2.4	-63.0 ± 2.6	-110.7 ± 11.0	-64.5 ± 6.2	-56.6 ± 13.6	0.156
Triglycerides, mg/dL	-19.4 ± 3.6	-17.7 ± 3.9	-20.3 ± 11.4	-27.5 ± 8.9	-46.9 ± 31.4	0.525
HDL cholesterol, mg/dL	2.4 ± 0.6	2.1 ± 0.6	-0.7 ± 2.2	4.9 ± 2.3	4.1 ± 4.0	0.493
Non-HDL cholesterol, mg/dL	-65.9 ± 2.4	-65.1 ± 2.6	-110.0 ± 11.4	-69.4 ± 5.4	-60.7 ± 12.4	0.190
Apolipoprotein B, mg/dL	-44.7 ± 1.3	-44.8 ± 1.4	-65.7 ± 8.5	-41.6 ± 3.7	-41.4 ± 9.1	0.280
Apolipoprotein A1, mg/dL	5.1 ± 1.2	5.3 ± 1.3	2.3 ± 6.1	2.4 ± 4.6	7.1 ± 8.4	0.908
Percent change						
LDL cholesterol, %	-44.9 ± 1.4	-44.5 ± 1.5	-57.7 ± 3.6	-48.1 ± 3.0	-44.7 ± 5.3	0.652
Total cholesterol, %	-29.6 ± 1.0	-29.3 ± 1.1	-42.2 ± 1.5	-30.7 ± 2.6	-27.8 ± 5.2	0.512
Triglycerides, %	-10.0 ± 2.4	-9.1 ± 2.6	-14.9 ± 8.2	-16.8 ± 7.6	-15.6 ± 9.9	0.801
HDL cholesterol, %	6.0 ± 1.1	5.5 ± 1.2	-0.9 ± 3.6	11.1 ± 4.2	9.8 ± 8.9	0.454
Non-HDL cholesterol, %	-40.7 ± 1.3	-40.2 ± 1.5	-53.7 ± 3.5	-44.7 ± 3.1	-39.5 ± 4.8	0.554
Apolipoprotein B, %	-38.8 ± 0.9	-38.8 ± 1.0	-47.5 ± 4.0	-38.8 ± 3.3	-33.8 ± 5.0	0.588
Apolipoprotein A1, %	4.7 ± 0.9	4.9 ± 1.0	2.9 ± 5.3	2.9 ± 3.3	6.7 ± 6.3	0.919

Variables are presented as the means ± SE. SE, standard error; ASCVD, atherosclerotic cardiovascular disease; LDL-C, low-density lipoprotein cholesterol; HDL, high-density lipoprotein.

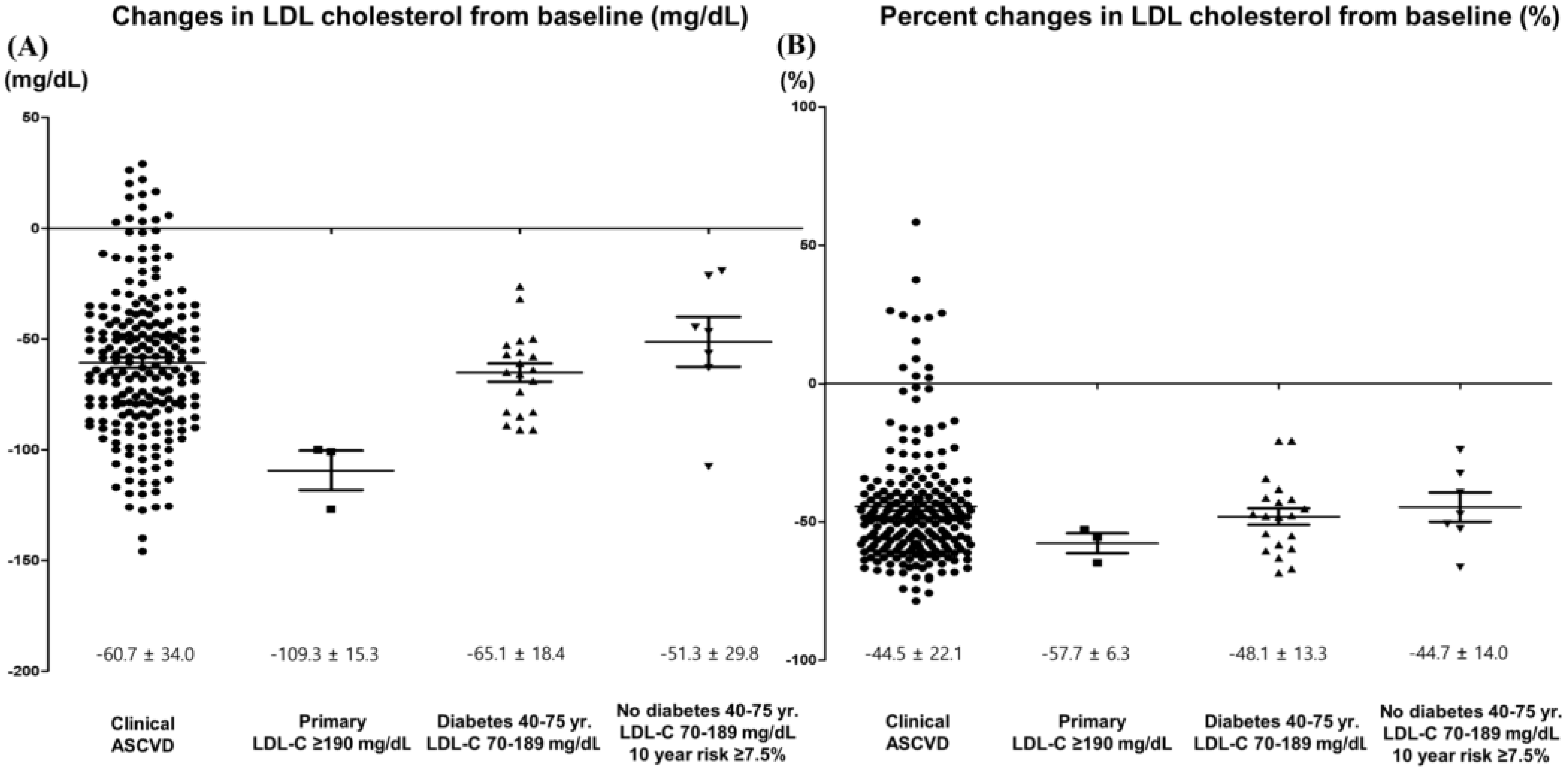

Supplementary Figure 1. Changes in LDL cholesterol per ACC/AHA 4 statin benefit groups. (**A**) Changes from baseline. (**B**) Percent changes from baseline. LDL, low density lipoprotein.
